# The Relationship Between Executive Functions and Academic Performance in Primary Education: Review and Meta-Analysis

**DOI:** 10.3389/fpsyg.2019.01582

**Published:** 2019-07-11

**Authors:** Alejandra Cortés Pascual, Nieves Moyano Muñoz, Alberto Quílez Robres

**Affiliations:** ^1^Department of Education Science, Faculty of Education, University of Zaragoza, Aragon, Spain; ^2^Department of Psychology and Sociology, Faculty of Humanities and Education, University of Zaragoza, Aragon, Spain; ^3^Department of Education, University of Zaragoza, Aragon, Spain

**Keywords:** executive functions, academic performance, primary education, relationship, meta-analysis

## Abstract

The purpose of this study was to research the relationship between executive functions and academic performance in primary education (6–12 years). Based on 21 samples (*n* = 7,947), a meta-analysis of random effects demonstrated a moderately significant weighted effect size (*r* = 0.365) and was found to be a good predictor of academic performance. For the subjects of language and mathematics, the results of the random effects model were similar and slightly higher for mathematics (*r* = 0.350; *r* = 0.365). Thus, the theory that executive functions have greater influence on mathematical performance is supported, especially in aspects such as coding, organization, and the immediate retrieval of information. Regarding the different executive function components (working memory, inhibition, cognitive flexibility, and planning), working memory had the highest presence (*k* = 14, *n* = 3,740) and predictive weight for performance, with an effect size of *r* = 0.370 for random effects, with a moderate level of significance. The moderating effect of variables such as gender and age were also analyzed. After performing a meta-regression, gender resulted in a value of *R*^2^ = 0.49; the age variable was not significant. This result is especially important since age has traditionally been considered to be the moderating variable of executive functions. The review reveals a good predictive power of executive functions in the primary education stage, and it is even higher at the early ages, indicating its great significance in describing future performance. The study also revealed the competencies and specific aspects of the executive functions that affect the way in which its components intervene in the academic area, demonstrating the mediating effect of variables such as physical fitness, motor skills, and memory processes.

## Introduction

The educational community has traditionally been interested in what is known as academic performance. This outcome is closely related to the teaching-learning process focused on a specific objective—achievement in school (Fleischhauer et al., 2010; Von Stumm and Ackerman, 2013). The topics of success or failure in school, discouragement, and dropping out of school have produced a great deal of research activity (Covington, 2000; Balkis, 2018). This interest is reflected in the study by Nieto (2008), who reviewed 654 studies conducted from 1970 to 1990. The author highlights how the studied variables that condition academic success in primary education have changed over time. In addition, the new century has seen the emergence of new variables that are original and methodological in nature such as group collaboration, collaborative work, project-based learning, and the continuous school day. The literature has traditionally categorized these variables as contextual or personal. The first group of variables includes socio-environmental (family, friends, colleagues), institutional (school, school organization, teachers) and instructional (content, methods, tasks) variables. The second group includes cognitive (intelligence, learning styles) and motivational (self-image, goals, values) variables (Zeegers, 2001; Vermunt and Endedijk, 2011). Therefore, if academic performance is “a construct that can have quantitative and qualitative values, and these values provide some evidence and a profile of the skills, knowledge, attitudes and values developed by the student in the teaching-learning process” (Edel, 2003, pp. 15–16), then brain functions are essential to understanding how this process unfolds. The findings of neuropsychology in this area are very useful for explaining this relationship (Kolb and Whishaw, 2007; Rosen et al., 2018). Therefore, according to Sesma et al. (2009) and Zelazo and Carlson (2012), educational research should focus on executive functions as they are fundamental for language development and thus for literacy (the foundation for learning) as well as for the processing and organization of received information.

Executive functions are understood as the distinct, but related, higher-order neurocognitive processes that control thoughts and behaviors aimed at achieving an objective or goal (Anderson, 2002; Zelazo and Carlson, 2012). Therefore, they regulate behavior and cognitive and emotional activity by means of a set of adaptive capabilities. These functions include working memory (the ability to temporarily manipulate information), inhibition (impulse control), cognitive flexibility (the ability to generate different solutions to a problem) and planning (the development of strategies to achieve an objective); the preceding functions are all considered to be basic processes of this variable (Baddeley, 1996; Anderson, 2002). Miyake et al. (2000) produced another similar classification that distinguished between working memory, inhibition and flexibility. Some of the research has produced evidence indicating that the components of this factorial structure are different and change with age (Willoughby et al., 2010; Lee et al., 2013; van der Ven et al., 2013).

Human memory has been one of the most studied constructs by psychologists (Loftus and Loftus, 2019). If the concept of memory represents the ability to store, retain and recall information, working memory or operational memory refers to storage that is short-term, temporary, and with limited capacity; it is also sensitive to distractions that enable the simultaneous performance of tasks (Baddeley, 2000). Its function is to retain information and manipulate it to perform a task or solve a problem. It receives only the information that a selective awareness recognizes as relevant and useful for performing the activity at hand. In addition, working memory is responsible for updating data and then manipulating and transforming them to plan and guide behavior in crucial cognitive processes such as language comprehension, reasoning, and mathematical calculation (Anderson and Reidy, 2012). Memory is thought to be modular, instead of unitary (Ferbinteanu, 2018). Therefore, memory processes are carried out by three coordinated modules: the phonological loop (responsible for manipulating auditory-verbal information), the visuo-spatial sketchpad (linked to visual and spatial information), and the central executive (responsible for the control of memory systems in directing attention to each task that must be performed and monitoring any changes in context) (Alexander and Stuss, 2000). Therefore, working memory is a multifactorial, short-term mnesic system that is prominently involved in the processes that regulate and coordinate the functions of executive control and selective attention and that are involved in problem-solving (Engle et al., 1999; Baddeley, 2000; Engle, 2002; Wilhelm et al., 2013).

Another component of executive functions, as noted by Matthews et al. (2005), is inhibition or behavioral control, which is the ability to suppress impulsive behaviors; that is, the ability to suppress dominant but irrelevant responses and focus on important information. One could say that inhibitory control moderates behavior, suppresses impulsive reactions to a stimulus, and enables an appropriate and thoughtful response. It allows individuals to make a choice about their own reactions and behaviors—to think before acting. Because this executive component has both behavioral and cognitive aspects, it can be understood in terms of behavioral inhibition (linked to motor control) and cognitive inhibition. The latter's impact on executive functions enables planning, analyzing and choosing the most appropriate response (Anderson, 2002). Therefore, “inhibitory control involves being able to control one's attention, behavior, thoughts, and/or emotions to override a strong internal predisposition or external lure, and instead do what's more appropriate or needed” (Diamond, 2013, p. 136).

Cognitive flexibility refers to quickly reconfigure the mind and to switch between tasks (Braem and Egner, 2018). It involves creating and choosing innovative work strategies (linked to creativity) from a variety of alternatives for performing a task but also the ability to modify the action plan depending on the conditions at any given time (Anderson, 2002; Cragg and Chevalier, 2012). Coulson et al. (2012) state that the need to approach complex problems from different points of view validates this theory of flexibility. There is evidence that the solution to a problem sometimes requires a broader and more creative vision to correctly implement the solution. Some authors such as Decety and Sommerville (2003) and Eslinger and Grattan (1993) recognize two aspects of this variable: on one hand, it is reactive in its ability to provide varying answers; on the other hand, it is spontaneous due to the wide range of ideas produced when faced with a new task.

Lastly, Anderson (2002) understands planning as the foresight to execute a task correctly and apply appropriate strategies. In the context of executive functions, planning refers to problem resolution, although as noted by Baddeley (1996, 2000), the working memory and the central executive must function properly to enable the ability to think about what should be done and to set priorities for action. However, planning goes further by coordinating these isolated processes in a certain way; an objective is set, the information is analyzed, the strategies that must be applied are selected, and the activities required to achieve the objective are assessed. Thus, achieving academic success is about effectively completing the important and necessary process executed by the executive functions by identifying the problem, defining the problem, finding alternative solutions, and developing an action plan (Anderson, 2002).

One view of academic performance defines it as the “level of knowledge demonstrated in an area or subject compared to the norm for the particular age and level of education” (Jiménez, 2000, p. 33). In addition, “it is the sum of distinct and complex factors that act in the person who is learning” (Garbanzo, 2007, p. 46). This construct refers to the evaluation of knowledge acquired in a school setting. It is dynamic in nature (the process of learning) as well as static (the product of learning) (Suazo, 2007). Therefore, it is presented as an index that assesses the quality of education, its efficiency and its productivity. It is the reflection of the different stages of an educational process whose objective is academic success, a process that is the focus of all the initiatives and efforts of educational authorities (Maturana, 2002). Currently, there is a general consensus in the scientific community on the existence of multiple variables and factors that explain academic performance, which display the complex and interdependent relationship between cognitive ability and emotion-attitude variables (Miñano and Castejón, 2011; Núñez-Peña et al., 2013). Another classification proposed by Passolunghi and Lanfranchi (2012) distinguishes between domain-general capabilities (the cognitive system as a whole) and domain-specific capabilities (which process a particular type of information). Domain-general capabilities notably include cognitive abilities (knowledge) and emotional skills that broadly predict school performance. Domain-specific capabilities (inferential skills, prescriptive processes) include skills that predict future performance in specific fields (development of a competency).

There are numerous articles that relate executive functions to academic performance (see Ahmed et al., 2018; Gordon et al., 2018). Studies such as those by Best et al. (2011), Castillo et al. (2009), and Ostrosky-Solis et al. (2007) conclude that working memory, a main component of the executive functions, is important for academic performance during the first few years of primary school. This variable develops rapidly at a young age and plateaus during adolescence. Align with this, a longitudinal study conducted by Ahmed et al. (2018) indicates that working memory at 54 months significantly predicts working memory at 15 years old. Furthermore, Tsubomi and Watanabe (2017) found that visual working memory, with and without distraction, develops until the age of 10. The study by Hall et al. (2015) on children 5 to 8 years old concluded that primary memory capacity improves with age. In addition, López (2013) study on third grade students found that good academic results in language and mathematics are related to this variable. Therefore, there is clear evidence that memory is a good predictor of academic performance by primary school students. However, this is not the case for the later stages of education because the predictive power of this variable diminishes at around the age of 12. Other authors in this line of research are Aronen et al. (2005), Best et al. (2011), Lee et al. (2009), and St. Clair-Thompson and Gathercole (2006). In addition, original results from Alloway et al. (2010) and Bull et al. (2008) indicated that the association of these variables is sustained over time, emphasizing the specific relationship between visuo-spatial working memory and performance in mathematics (domain-specific); the other executive components predict domain-general learning. Focusing on another facet, studies by Alloway et al. (2008) and Abreu et al. (2014) concluded that learning difficulties are explained by deficiencies in this executive component and are therefore reflected in academic performance.

Various studies have focused on the analysis of other components of the executive functions. For example, behavioral inhibition, that is self-control is shown to be relevant for academic achievement (Duckworth et al., 2019). In a longitudinal study of children 3 to 7 years old, Blair and Razza (2007) found that the relationship between academic performance and attention control and inhibition depend on age and the subject studied. Latzman et al. (2010) studied whether different academic subjects place specific demands on the various components of executive functions, analyzing the link between this variable and the performance of children 11 to 16 years old in science, mathematics, social studies, and reading. Of the various factors studied, cognitive flexibility was associated with reading and science and the control or regulation of reading and social studies capabilities. Gerst et al.'s (2017) study of children 5 to 11 years old found that inhibition and planning were the strongest predictors of mathematical calculation. For Sesma et al. (2009), working memory and planning are needed more when the complexity of a written text increases, and inhibition is related to mathematics and science. As such, these results suggest that there are specific demands placed on the various executive functions depending on the academic domain (Passolunghi and Lanfranchi, 2012). Therefore, there is widespread agreement that the skills related to executive functions, such as recalling and retaining information (working memory), the ability to suppress distractors (inhibition control-attention control), the ability to combine different tasks (cognitive flexibility), and planning (the ability to foresee the correct execution of a task) are essential for academic achievement since changes to these skills decrease the likelihood of success.

The current study analyzed the relationship between executive functions and academic performance in primary education. This was considered necessary as most of the publications on academic performance in primary education over the last decade have found this variable to be more significant for academic performance than the intelligence quotient, the variable traditionally considered to be the best predictor of academic success (Ren et al., 2015). In addition, we studied which executive function component (working memory, inhibition, cognitive flexibility and planning) would have a greater predictive weight since most of the existing studies have found a single component in the 2 to 6 years old age group (Wiebe et al., 2008) and a multifactorial composition after the age of seven (Jarvis and Gathercole, 2003; Jacobson and Pianta, 2007). It was also important to study whether the executive functions were included within the domain-general or domain-specific cognitive variables, whether their components changed according to the academic subject, whether they predicted performance in specific competencies (Im-Bolter et al., 2006), and whether they have a moderating function in other variables regarding academic performance. Lastly, we analyzed possible moderating variables such as sex or age. Age has traditionally been the variable with the moderating effect. However, this result was not expected for this study as it focuses on primary school students 6 to 12 years old, a group in which males and females have different levels of maturity. Data were obtained for all these study objectives to calculate the effect size of the relationships and the significance of the variability between the samples.

Based on this literature review, our research questions are aimed to explore whether there is a relationship between executive functions and academic achievement among students from Primary education. Also, we will take into account whether this association is influenced by the following aspects: subject –e.g., mathematics, literature…-, gender, and age. For this background, our research questions are about the relationship between executive functions and academic performance in the stage of Primary Education. In addition, a specific study is carried out on this relationship and specific areas such as language and mathematics taking into account other variables such as gender and age.

## Methods

### Inclusion and Exclusion Criteria

The following inclusion criteria were established: (a) the studies should provide clear and correlational statistical data between the variable of executive functions or any of its components (working memory, inhibition, flexibility or attention) and academic performance; (b) age, since the research focused on primary school students 6 to 12 years old; (c) articles that studied the same variables from an inverse approach, that is, the relationship between the executive functions and poor academic performance; (d) articles that included in their samples any individuals diagnosed with a DSM-5 mental disorder and did not exclude individuals with normal development; (e) articles that researched samples of individuals with low socioeconomic status; and (f) longitudinal studies conducted in the pre-school stage that focused on predicting future performance and those that started in primary school and progressed through secondary school (17 years). The following criteria were grounds for exclusion from the study: (a) studies conducted in a clinical context –that is, in samples with a typical development-; (b) studies where the entire sample consisted of individuals diagnosed with learning disorders; and (c) studies that failed to fulfill the criterion of statistical clarity. The reason for this last exclusionary criterion is that, per Chalmers et al. (2002), the individual studies had to be integrated into the current study to conduct the analysis; in addition, they had to have a certain degree of similarity and comparability.

### Search Strategies

**Figure 1:**
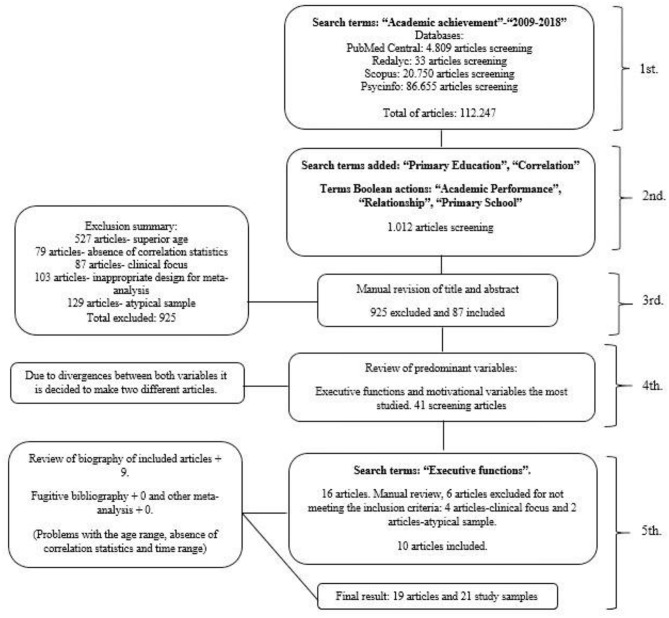
Flowchart of the inclusion protocol.

An electronic search was conducted (July–September 2018) on the Scopus, PsycINFO, PubMed Central and Redalyc databases. The search was performed in the English language and applied the terms “academic achievement,” “primary education,” and “correlation” with a 2009–2018 date range. This filter yielded a total of 1,012 documents that met the search requirements. Next, the titles and abstracts of these articles were reviewed, and 925 were excluded because they corresponded to clinical settings, did not meet the age parameters, followed a non-descriptive methodological approach, or did not offer clear statistical data. The final sample of 87 publications provided information on the variables that were studied the most over the last decade and that were related to academic performance. The most numerous group of articles studied the executive functions and personal motivation factors (41 articles). Considering the divergence between these two topics, it was decided that each would be studied separately, focusing first on the executive functions. An in-depth review of the material selected reduced the number of valid studies to 10 that could be used in the proposed research. This number was considered to be insufficient; thus, the bibliographic references in the articles were reviewed, and those not meeting the language criterion were eliminated in the search scope. A search of the “gray” or “fugitive” literature (Cooper et al., 2009) was also conducted, which included conference databases, doctoral theses, conferences, and meetings. These publications did not yield any information of interest for the present study; however, it was not an exhaustive search. Ultimately, 19 articles were selected (19 in English and 1 in Spanish) that provided 21 samples or databases (Alloway and Alloway, 2010 and Hall et al., 2015 provided two each) for use in a meta-analysis of the predictive capacity of executive functions in the academic performance of primary school students. In addition, the executive function components that recurred in the analyzed studies were working memory, inhibition, cognitive flexibility, and planning. No additional data beyond those published were requested from any author. The current research study will become part of a section on logical reasoning, verbal factors and working memory to be included in a doctoral thesis that consists of a compendium of publications titled “Variables that Influence Academic Performance in Primary Education: Tradition or Innovation” (Figure 1).

### Coding Procedure

The study complies with the guidelines from the manual of systematic reviews (see Cochrane 5.1, point 1.2.2, Higgins and Green, 2008), in which it is established a set of clear objectives, specific search terms and eligibility criteria for previously defined studies. All studies were coded separately. In some articles, the executive functions are referred to as a single factor, and others refer to the different factors that compose them (working memory, inhibition, cognitive flexibility, and planning). Academic performance was measured in two dimensions: reading, measured in selected studies such as fluency and reading accuracy by reading words (reading comprehension, reading fluency, vocabulary) and mathematics (mathematical reasoning, calculus, arithmetic). A total of 198 effect sizes were coded using the correlation itself as a reference, and the corresponding standard error and confidence intervals were calculated. Similarly, these data were integrated using averages and weighting, and the academic performance and the overall executive functions of each study were calculated so that statistical analyses could later be performed individually.

### Effect Size

This study's statistical approach applied an analysis of two continuous variables; thus, the correlation coefficients were used as the effect size to establish the relationship between executive functions and academic performance. Regarding the sample correlation coefficients, it was decided to transform them into Fisher *Z*-values, thus ensuring that the variance of the effect size will be based on the sample size. Cohen (2013), an effect size is considered to be small when the correlation coefficients do not exceed 0.10; they are considered to be moderate at 0.30; and they are considered to be large if they exceed 0.50.

### Statistical Analysis

To examine the variability of the sampling, the parameters studied were the Cochran *Q* test (to test the null hypothesis of homogeneity between the studies) and the *I*^2^ (proportion of the variability). According to Higgins et al. (2003), if *I*^2^ reaches 25%, it is considered low; It is considered moderate if it reaches 50%; and it is considered high if it exceeds 75%. This may be due to a sampling error, a real variability in the variance and the size of the effect, or the influence of a third variable acting as a moderator. In this sense, different meta-analyses were applied that excluded studies with atypical data. In addition, the results produced by model 1 (fixed effects) and model 2 (random effects) were analyzed. Due to the number of research studies in the sample (21 databases), it was determined that model 1 is initially more appropriate (Overton, 1998; Schulze, 2007), since a study of fixed effects assumes a real and real size of the effect, and the variability of the sampling supposes an error in the sampling. However, in a random effects model with a more conservative approach, the sampling variability is lower and is not considered a sampling error but a real variability in the variance and in the size of the effect (Borenstein et al., 2011). Therefore, considering both approaches, we decided to perform an initial analysis that included all the studies and then eliminate those that showed outliers. The comparison of the two showed that the sampling variability was affected by some of them (*Q* = 119.359, *I*^2^ = 83.24); therefore, they were treated separately to explain the results. To clarify, these research studies were not eliminated, and although the sampling variability decreased (*Q* = 43,536, *I*^2^ = 58,655), even without them, the sample variability did not reach 50%. Therefore, in the presence of variability and heterogeneity, it is established to follow the work based on the random effects model. In addition, a meta-regression of random effects was carried out, taking gender and age as moderators, since numerous studies indicate that executive functions and their cognitive influence vary with age (Ostrosky-Solis et al., 2007; Castillo et al., 2009). The software used to classify and encode data and to produce descriptive statistics was the EZAnalyze add-on (Microsoft Excel, 2007). The integral meta-analysis software (CMA, Biostat, USA) was used for meta-analysis and meta-regression calculation data.

## Results

### General Description of the Studies Included in the Research

A search of the literature related to the topic and published in the last decade produced a small number of articles. This is because the search was limited to a specific age range (6 to 12 years) corresponding to primary education. In addition, there was a requirement that the studies have clear correlation statistics so that the data could be integrated. The studies describe research conducted in various parts of the world, which enabled us to determine if the results displayed significant differences depending on the dominant culture in the respective countries. Therefore, we found not only diverse cultures but also a variety of educational systems, although it should be noted that the African continent and South Asia were not represented. Of the 21 databases pertaining to the 19 articles selected, a total sample of 7,947 individuals was obtained (Table 1). The smallest data set contained only 60 individuals, and the largest data set contained 2,036. Three of the studies did not provide information on the number of participants by gender or on the composition of the sample; the published data indicate that 51.27% of the individuals were male and 48.73% were female. A total of 26.31% of the studies pertained to the United States, representing 45.72% of the participants. The Netherlands had 15% of the studies, representing 25.64% of the participants. The United Kingdom had 15% of the articles but only 5.78% of the participants. The sole Norwegian study stands out with 14.16% of the sample. Therefore, Europe represents 48.55% of the sample, North America 48.28%, South America 1.44%, and Asia 1.73%.

**Table 1:** Characteristic of studies included in the meta-analysis.

	**References**	**Country**	**Population**	**Males**	**Females**	**Age**	**Performance measurement**	**Other variables included**	**Executive functions specified**	**Distribution of participants**
**1**	Aadland et al., 2017	Norway	*n =* 1,129	588	541	10–11 years	Arithmetic, reading and English	Aerobic capacity, motor skills, arithmetic, reading	Working memory, inhibition, and cognitive flexibility	57 schools in a southern county with more than 100,000 inhabitants
**2**	Aarnoudse-Moens et al., 2013	The Netherlands	*n =* 200 *n =* 230	212	218	4–12 years	Mathematics	Mathematics (standardized tests), attention (parents and teachers)	Verbal and visuo-spatial working memory, impulse planning, and control, verbal fluency	Three schools in the same city
**3**	Alloway and Alloway, 2010	United Kingdom	*n =* 98	50	48	5–11 years	Mathematics and language	IQ, reading comprehension, reading, spelling, mathematical reasoning, and numerical operations	Working memory and short-term verbal memory	No information
**4**	Best et al., 2011	United States	*n =* 2036	unknown	unknown	5–17 years	Mathematics and reading	Identify letters and words, comprehension, vocabulary; calculation, applied problems, and quantitative concepts	Executive functions: planning, attention, simultaneous, and successive	Entire country
**5**	de Bruijn et al., 2018	The Netherlands	*n =* 472 *n =* 473	208/210	264/263	7–9 years	Mathematics and spelling	Physical fitness, mastery of mathematics and spelling	Inhibition, verbal and visuo-spatial working memory, change of attention	12 schools in the northern part of the country
**6**	Bryce et al., 2015	United Kingdom	*n =* 66	37	29	5–7 years	Mathematical reasoning and reading of words	Mathematical reasoning and reading of words	Inhibitory control, working memory	Six schools in Bristol (United Kingdom)
**7**	Abreu et al., 2014	Brazil	*n =* 106	60	66	6–8 years	Reading	Reading, writing, mathematics, spoken language, natural and social sciences	Cognitive flexibility, working memory, inhibition, selective attention.	Various populations, various schools, urban environment
**8**	Gerst et al., 2017	United States	*n =* 93	41	52	5–11 years	Reading comprehension and mathematical calculations	Conduct, working memory, reading comprehension skills, mathematical calculations	Working memory, inhibition and change	Three schools in a large metropolitan area
**9**	Gómez-Veiga et al., 2013	Spain	*n =* 77	39	38	8–9 years	Reading comprehension	Spelling and reading comprehension; updating and retaining information; and non-verbal intelligence	Working memory	Two schools in a population of more than 100,000 inhabitants
**10**	Gray et al., 2015	Canada	*n =* 204	101	103	5–9 years	Mathematics and reading	Addition, subtraction, calculation, and reading ability, and fluency	Lack of attention and working memory	Large, suburban and rural school district, Southern Ontario
**11**	Hall et al., 2015	United Kingdom	*n =* 101	50	51	5–8 years	Mathematics and reading	Reading, comprehension of sentences, procedures, problem-solving, and reasoning	Primary memory	No information
**12**	Mulder et al., 2017 (44 children of unknown gender)	The Netherlands	*n =* 552	236+22	277+22	3–6 years	Literacy, mathematics	Knowledge and estimation of numbers, mathematical skills, knowledge of letters, phonological awareness, language, reading-writing skills	Selective attention, visuo-spatial working memory, short-term visuo-spatial memory, short-term verbal memory	Entire country
**13**	Oakhill et al., 2011	United Kingdom	*n =* 97 *n =* 100	unknown	unknown	6–11 years	Reading accuracy and comprehension	Cognitive skills: verbal, numerical, spatial; reading (accuracy, comprehension) and working memory: verbal, numerical and spatial	Working memory	Five schools in the country
**14**	Oberer et al., 2018	Switzerland	*n =* 134	66	68	5–9 years	Mathematics and reading	Sequences, addition-subtraction, comprehension and reading speed, visual-motor coordination and, physical fitness	Inhibition, changing and updating	Schools in a college town
**15**	Ribner et al., 2017	United States	*n =* 1292	unknown	unknown	5–12 years	Mathematics and reading	Counting, measuring, arithmetic, verbal and non-verbal operations, letters, words, reading ability	Working memory, inhibitory control, switching attention	Two geographical areas of the country with high poverty rates
**16**	Sánchez-Pérez et al., 2018	Spain	*n =* 142	74	68	6–12 years	Mathematics and reading skills	Study skills (organization and habits); applied calculations and problems; letter sounds, word reading, intonation, etc.; social skills	Activation control, focus of attention and inhibitory control.	Two geographical areas of the country with high poverty rates
**17**	Sesma et al., 2009	United States	*n =* 60	30	30	9–15 years	Reading comprehension	Attention, decoding, fluency and vocabulary	Working memory and planning	No information
**18**	Tsubomi and Watanabe, 2017	Japan	*n =* 121	67	54	7–12 years	Literacy (reading and writing), mathematics, science, music, art, physical education	All subjects assigned	Visual working memory	One school
**19**	Welsh et al., 2010	United States	*n =* 164	71	93	4–6 years	Beginning literacy and basic arithmetic	Reading skills, arithmetic skills and cognitive skills	Working memory and attention control	Schools in three Pennsylvania counties

It is interesting to note that five articles address only the reading aspect of academic performance; one article addresses only the mathematical aspect, and the rest examine both reading and mathematical skills. Furthermore, of the executive functions, working memory is the factor that appears most often, sometimes in conjunction with inhibition, flexibility or attention. Seven of the studies have a longitudinal design, and two address academic performance from the opposite perspective (poor reading performance). Of these, several stand out: Aarnoudse-Moens et al.'s (2013) study on the effect of premature birth on subsequent performance with a control group, and Sesma et al.'s (2009) studies on groups diagnosed with conditions such as ADHD, dyslexia, and dyspraxia. Sesma et al.'s study examines a group with weak word recognition and another with poor reading comprehension. The socioeconomic status of the families, the educational level of the parents, language difficulties, gender, and age all are examples of the diverse interests represented in these articles. The sources of the samples vary; some were obtained from existing projects, whereas others pertain to the entire country, to a single city, to a single school, to rural areas, or to urban areas (Table 1).

The procedures followed to measure academic performance for the most part correspond to the standard achievement tests of each country. The Woodcock-Johnson III test was used by five authors including Welsh et al. (2010) who also used the TOPEL test for reading achievement. Sánchez-Pérez et al. (2018) opted for PROLEC. The WIAT-II tests were selected for the studies by Bryce et al. (2015) and Sesma et al. (2009). Only Alloway and Alloway (2010) used the Wechsler test, and Oakhill et al. (2011) used the Neale Analysis of Reading Ability (NARA) and CAT tests. The studies by Tsubomi and Watanabe (2017) and by Abreu et al. (2014) were exceptions in that the teachers themselves were responsible for evaluating the academic performance of the students. In any case, it is indicated that the reading tests used are aimed at reading words to measure fluency and accuracy. As for the instruments used to measure the executive functions, sometimes a single component was measured such as working memory, etc., and others considered the executive functions as a whole, always depending on the age of the subjects being evaluated. When working memory was addressed, the applied tests were the “Automated Working Memory Assessment-AWMA” (Alloway et al., 2008) and the Wechsler Intelligence Scale for Children (WISC-III and IV). The Stroop Color test was used in the studies on inhibition, and a wide variety of other instruments were used for other components (for example, the duck task for cognitive flexibility or the Tower of London for planning); the use of computers and specific software for these tests was noteworthy.

### Effect Size and Statistical Significance

**Figure 2:**
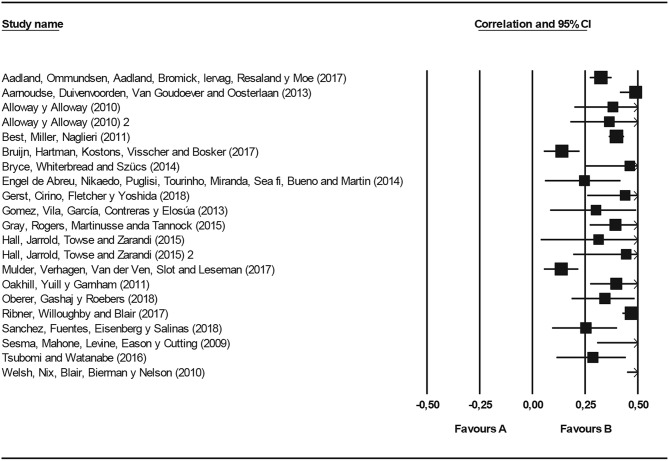
Forest plot effect size (Pearson's r). Executive functions—academic performance.

Figure 2 (Forest Plot) and Table 2 both present the effect size and confidence interval (95%), for the studies with regard to general academic performance and overall executive function. The individual analysis of each sample is presented as well as the weighted results for random effects model. The meta-analysis of the variables concludes that the data obtained have good consistency. The executive functions presented an effect size of *r* = 0.365, with a 95% confidence interval ranging between 0.309 and 0.419 for a sample of *k* = 21 and a population of *n* = 7,947. None of the intervals were zero; as such, there is a medium weighted mean effect size with a significance of *p* < 0.05. A second calculation (for most of the studies linking academic performance to mathematics and language) presents the effect size and the confidence intervals of the executive functions for the two academic areas in Table 3. The results indicate that the effect size for mathematics is slightly higher (*r* = 0.365), which is consistent with other studies, indicating that executive functions are a better predictor for this area than for language (Brock et al., 2009; Willoughby et al., 2012). Again, there is a medium weighted mean effect size with a significance of *p* < 0.05.

**Table 2:** Effect size: executive functions—academic performance.

		**Effect size and 95% interval**		
**Model**	***K***	**Point estimate**	**Lower limit**	**Upper limit**
Random	21	0.365	0.309	0.419

**Table 3:** Effect size: executive functions—academic performance in mathematics and language.

		**Effect size and 95% interval**		
	***K***	**Point estimate**	**Lower limit**	**Upper limit**
**MODEL FOR MATHEMATICS**				
Random	18	0.365	0.304	0.422
**MODEL FOR LANGUAGE**				
Random	20	0.350	0.287	0.409

Next, the same procedure was performed for the executive components. Working memory is the factor that is most prominent in the research (in 14 of 21 databases). As such, it was the first factor analyzed with respect to overall academic performance and subsequently with respect to mathematics and language (Table 4). The effect size for this first statistical calculation is 0.370 for random effects, with a confidence interval of 95% (0.287 to 0.447). The sample consisted of 13 studies with *k* = 14 databases and a population of *n* = 3,740 individuals. Eleven articles studied the links between performance in mathematics and working memory, and 13 studied the links to language development. A moderate and average effect size was found for both studies. These results support the theory that the executive functions are a better predictor of performance in mathematics, especially in aspects such as coding, organization and the immediate retrieval of information—what we call working memory (Bull and Scerif, 2001; St. Clair-Thompson and Gathercole, 2006).

**Table 4:** Effect size: working memory—academic performance, performance in mathematics and language.

		**Effect size and 95% interval**		
	***K***	**Point estimate**	**Lower limit**	**Upper limit**
**MODEL FOR MATHEMATICS**				
Random	11	0.374	0.287	0.454
**MODEL FOR LANGUAGE**				
Random	13	0.331	0.245	0.411
**MODEL**				
Random	14	0.370	0.287	0.447

### Heterogeneity Analysis

The variability among the different samples of the relationship between academic performance and executive functions was significant (*Q* = 119.349, *df* = 20, *p* < 0.000), and the *I*^2^ was 83.242%), which was higher than expected. However, the results of the random effects model were more conservative (since there were fewer than 30 samples). With these results, it was appropriate to test the sensitivity of the sample by performing a second meta-analysis that excluded two studies: de Bruijn et al. (2018) and Mulder et al. (2017). This second meta-analysis yielded the following: *Q* = 43.537, *df* = 18, *p* < 0.001, *I*^2^ of 58.656%, and significant moderate variability. Two findings stand out: the first is the outlier values obtained by the discarded studies, and the second is the possible existence of moderating variables; these will be specifically addressed in another section. The meta-analysis performed for the variables of academic performance and working memory presented similar results (Table 5): *Q* = 87.910, *df* = 13, *p* < 0.000 and *I*^2^ of 85.212%; these values again decreased upon excluding the three above-mentioned articles.

**Table 5:** Sampling variability: working memory—academic performance.

	**Heterogeneity**			
**K**	***Q***	***df***	***p***	***I^2^ (%)***
14 ^*^12	87.910 ^*^23.628	13 ^*^11	0.000 ^*^0.014	85.212 ^*^53.445

* *Excluding outlier data*.

### Atypical Values or Different Sample Sizes

Once the studies with outlier values (Mulder et al., 2017; de Bruijn et al., 2018) were identified, and although the sampling variability was diminished by excluding them, there were no significant differences in effect size (*r* = 0.365, *Q* = 119.349, *df* = 20, *I*^2^ = 83.242%; *r* = 0.398, *Q* = 43.537, *df* = 18, *I*^2^ = 58.656%). Furthermore, when studies with larger populations were discarded, there were practically no differences in the results (*r* = 0.359, *Q* = 85.318, *df* = 17, *I*^2^ = 80.075). Therefore, we decided not to exclude any sample from the meta-analysis since no sample amounted to 50% of the statistical weight. In addition, the differences in effect size were not significant. However, distinct analyses of the three studies were conducted to explain the causes of this reduced variability of the differences indicated by the values for the *Q*-statistic.

### Publication Bias Analysis

**Figure 3:**
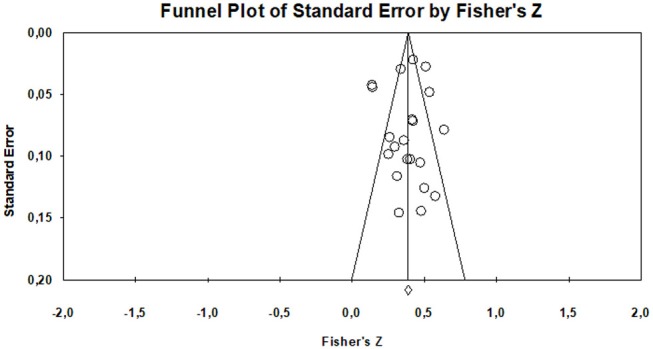
Funnel plot. Executive functions—Academic performance.

The funnel plot (Figure 3) facilitates the verification of the existence or not of bias regardless of the size of the sample. This graph shows that the results obtained for the Z values from the studies included in this meta-analysis, show small values that range between 0 and 1. As indicated by Palma and Delgado (2006) they would indicate an absence of bias, since that the existence of the same is considered from the significantly distant values of 0. In the same way, when the Egger test is performed, the value in the interjection point of the ordinate axis is 0.24 (close to 0). This author points out that a higher value would indicate the existence of bias (Egger et al., 1997). At the same time, the *p*-value (0.404) is therefore >0.1, so the results of the funnel plot would be confirmed. From all these data it is deduced that the present study does not show any problem related to a possible publication bias.

### Moderating Variables

Various studies confirm that both age and gender are two moderating variables of executive functions (Ostrosky-Solis et al., 2007; Castillo et al., 2009; Ganley and Vasilyeva, 2011; Rogers et al., 2011; Bull et al., 2013; López, 2013). Therefore, these variables were analyzed to check their degree of moderation or their power to explain the variance. First, a meta-regression (over random effects) was performed on age as a moderating variable, and no significance was found. A possible explanation for this lack of significance is the age parameter because primary education covers a range of young ages linked to a specific psychosocial stage of development. A second meta-regression (over random effects) was performed that included gender, and moderate significance was found (*R*^2^ = 0.49); that is, gender can explain 49% of the variance. Unlike the studies referenced above, the female gender explained this relationship, possibly because of the greater tendency in females toward mature development (Ausubel and Sullivan, 1983); as such, this question was deferred to a future research study. Regarding this model's goodness of fit for the modified sample, the results (*Q* = 54.16 and *I*^2^ = 66.77%) were lower than those of the meta-analysis due to the changed values in the meta-regression. In conclusion, of all the possible moderating variables in the meta-analysis, only gender had the capacity to explain a moderate degree of variance (49%). No significance was found for the age variable because of the sample homogeneity that occurs when researching a specific period of education (see Tables 6, 7).

**Table 6:** Meta-regression with moderating variables: Age and Gender.

**Summary of models: random effects (MM), Z-distribution, fisher's Z**									
				**Test of model (a)**			**Goodness of fit (b)**		
	**Model name**	**TauSq**	***R^2^***	***Q***	***df***	***P-value***	***Q***	***df***	***P-value***
(Without moderators)	Model 1	0.015	0.000	0.000	0.000	1.000	119.350	20.000	0.000
(Gender)	Model 2	0.008	0.490	8.540	2.000	0.014	54.160	18.000	0.000
(Age)	Model 3	0.017	0.000	0.020	1.000	0.655	110.420	19.000	0.000

**Table 7:** Meta-regression: Gender.

**Main results for model 2: random effects (MM), Z-distribution, fisher's Z**							
**Covariate**	**Coefficient**	**Standard error**	**95% lower**	**95% upper**	***Z*-value**	**2-sided *P*-value**	**VIF**
**Intercept**	0.440	0.034	0.373	0.507	12.82	0.0000	1.757
**Male**	0.002	0.001	0.000	0.005	2.06	0.0396	48.028
**Female**	−0.003	0.001	−0.005	0.000	−2.34	0.0195	48.028
**Statistics for Model 2**							
**Test of the model: Simultaneous test that all coefficients (excluding intercept) are zero**							
*Q* = 8.54, *df* = 2, *p* = 0.0140							
**Goodness of fit: Test to determine if unexplained variance is zero**							
Tau^2^ = 0.008, Tau = 0.088, *I*^2^ = 66.77%, *Q* = 54.16, *df* = 18, *p* = 0.0000							
**Comparison of Model 2 with the null model**							
**Total variance between studies (intercept only)**							
Tau^2^ = 0.015, Tau = 0.124, *I*^2^ = 83.24%, *Q* = 119.35, *df* = 20, *p* = 0.0000							
**Proportion of total between-study variance explained by Model 2**							
*R*^2^ analog = 0.49							

## Discussion

The purpose of this study was to investigate the links between executive functions and academic performance in primary education over the last decade. This review and meta-analysis found that executive functions are considered to be good predictors of academic achievement in normally developed children (*r* = 0.365). Delving deeper, evidence of the following was obtained: (a) the multifactorial composition of the executive functions, in which working memory has the most significant influence on academic performance (*r* = 0.370); (b) the presence of a certain moderating effect of executive functions on other variables of academic performance; and (c) the moderating function of gender (*R*^2^ = 0.49).

The literature provides numerous examples of the importance of executive functions in achieving academic success (see Huizinga et al., 2018; Willoughby et al., 2019). Language development is essential for proper learning, and cases of low reading ability demonstrate some deficiency in these skills (Abreu et al., 2014). There is a recognized problem specific to language that is associated with a poor working memory and that prevents normal language development (Im-Bolter et al., 2006). Furthermore, distraction directly influences an individual's ability to focus on and correctly capture external stimuli (Gray et al., 2015). Therefore, if the verbal component and logical reasoning are the foundation for good academic performance, they are themselves related to the development of executive functions. In addition, if they have a robust power to predict subsequent academic success, there is ample evidence that justifies an interest in understanding and examining all aspects of their behavior with respect to academic performance.

The intelligence quotient has traditionally been the most important factor in predicting academic performance (Vukovic and Lesaux, 2013; Ren et al., 2015); however, it diminishes in importance at the university level (Reynolds and Walberg, 1992; Patrikakou, 1996). Some studies conclude that intelligence is the variable with the most variance in explaining school performance (Staff et al., 2014). The data gathered for this review and meta-analysis confirm that, at present, the executive functions and the intelligence quotient have the same degree of predictive capacity regarding school performance, with the intelligence quotient being more important for new learning, and the executive functions being more important for learning that is repetitive and focused on competencies. Therefore, our findings are in line with some recent research (Costa and Faria, 2018; Lotz et al., 2018). Particularly, for Aarnoudse-Moens et al. (2013), the “g” intelligence factor explains poor performance in mathematics during the pre-school years; however, they found similar prediction values for executive functions and the intelligence quotient during primary education. Ribner et al. (2017) obtained similar results, but with respect to good student performance in mathematics and language. These results suggest that mathematical problems are increasingly complex at this stage of education, which is why highly developed cognitive skills are necessary. The research by Best et al. (2011), Hall et al. (2015), or Tsubomi and Watanabe (2017) all highlight the importance of executive functions in the early years of primary education and the rapid development of working memory at a young age, to achieve stability between the ages of 10 and 12. In Alloway and Alloway's (2010) article, this mnesic-executive aspect emerges as a better predictor of future performance (in literacy and mathematical reasoning) than the intelligence quotient. In addition, they highlight the importance of early intervention to improve future results as well as the independence of both variables. These results are explained by the static nature of intelligence as opposed to the executive functions that change with age and neurocognitive maturation. Gómez-Veiga et al. (2013) performed a regression analysis and found that working memory (ß = 0.28) and fluid intelligence (ß = 0.30) explain 33% of the variance in reading comprehension. Similar predictive values are found in the direct correlations between the aspects of memory that are linked to executive functions and academic performance and the aspects of intelligence that are linked to academic performance.

Another important issue is the dilemma raised regarding the homogenous or multifactorial composition of the executive functions that are explained by their own evolution and development (Best et al., 2011). The results of this study support the notion of a multifactorial composition of executive functions within the context of primary education (ages 6–12) because the meta-analysis revealed that working memory is the most studied component (14 of 21 databases), displaying an effect size of 0.370 for random effects, which gives it more predictive power than inhibition. There are numerous studies in this field on pre-school children; however, there are few for the primary education years. There are two aspects to consider. On one hand, Wiebe et al. (2008) found that executive functions in children 2 to 6 years old have a homogenous composition. On the other hand is the opinion that this variable has several related but totally distinct components: working memory, inhibition and cognitive flexibility (Miyake et al., 2000; Bull and Scerif, 2001; St. Clair-Thompson and Gathercole, 2006). Some authors include another factor—planning (Anderson, 2002). Several studies (Isquith et al., 2004; Senn et al., 2004; Huizinga and van der Molen, 2007) conclude that inhibition is the best predictor of academic performance up to the age of seven. After that age, working memory is the most important, and then cognitive flexibility becomes the most important after the age of 11. These findings supposedly indicate that inhibition develops first, with other components emerging later such as working memory and cognitive flexibility. That is, age produces changes in the relationships between the executive function components and academic performance (Jarvis and Gathercole, 2003; Jacobson and Pianta, 2007).

This study sheds light on the question about the modularity of brain, that is, that the brain can be conceptualized as a network which comprises some modules (Baniqued et al., 2018). Therefore, this review of indicates that the executive functions have general, overall characteristics and their components have specific characteristics. The distinct factors of this variable are better related to academic performance depending on the subject matter studied. This is because the specific development of certain skills and abilities is needed for school performance. The results of this meta-analysis are consistent with the literature reviewed, and they highlight the relationship between mathematics and the visuo-spatial aspect of working memory. Moreover, most of the executive function components correlate better with academic performance in mathematics than in language (*r* = 0.374; *r* = 0.331). It can also be concluded that, despite its general nature, by contributing to the development of different aspects of learning, working memory becomes more specific in nature in the development of particular skills. Therefore, it is identified as being a relevant and specific sub-variable depending on whether its auditory-verbal or visuo-spatial aspect is engaged. Similarly, the other executive function components such as inhibition (with verbal or visual distractors), cognitive flexibility, selective attention (of distraction or attention with verbal or visual stimuli), and planning display specific characteristics by significantly correlating with the development of academic skills. de Bruijn et al. (2018) published a study on poor reading performance in which working memory became more important than inhibition. That is, when encountering learning problems, the variables act differently. Gómez-Veiga et al. (2013), Nouwens et al. (2017), Oakhill et al. (2011), and Sesma et al. (2009) all agree that the two aspects of working memory (visuo-spatial and auditory-verbal), are deemed to be predictors of reading comprehension, especially the relationship between the auditory-verbal aspect and the tasks of storage and symbolic recall. Furthermore, Tsubomi and Watanabe (2017) found that visual working memory without distractors selectively predicts performance in mathematics. The study by Welsh et al. (2010) presented results that demonstrate predictive reciprocity between mathematics and the executive functions of working memory and attention. For Aarnoudse-Moens et al. (2013), Gray et al. (2015), and Mulder et al. (2017), inhibition explains the lack of attention and highlights the visuo-spatial component of the memory function for performance in mathematics. In addition, Gerst et al. (2017) contend that cognitive flexibility and planning are good predictors of that area. Abreu et al. (2014) establish relationships between reading and the executive functions of working memory and cognitive flexibility.

It should be noted that in some of the articles reviewed in this study, the executive functions, in addition to acting as a predictor in direct models of academic performance, have a certain moderating influence on other variables such as physical fitness, motor skills or memory processes. The studies by Aadland et al. (2017) and de Bruijn et al. (2018) introduce the variable of activity or physical aptitude. The latter study examines its relationship with poor academic performance; however, both adopt the perspective of the moderating or mediating effect of the executive components. Aadland et al. (2017) did not find any potential moderating influence of executive function on physical activity and academic performance; however, they did find a slight effect on the ability to work with numbers and motor skills. From the opposite perspective, de Bruijn et al. (2018) demonstrated an indirect relationship between physical fitness and poor academic performance, moderated by the executive functions with respect to mathematics and spelling. In addition, verbal working memory is both a domain-general and domain-specific mediator, and its visuo-spatial aspect is related to poor academic performance in mathematics. Similarly, Oberer et al. (2018) find that executive functions, visual motor coordination, and physical fitness predict subsequent academic performance and that executive functions act as moderators between physical fitness and academic performance. Bryce et al. (2015) find that the variable of cognitive abilities robustly contributes to school performance within a structured model where the executive components act as mediators. All this is based on the close relationship between the two such that the performance of the former will be predetermined by the development of the latter. They conclude that executive functions contribute positively to enabling the youngest students to use their cognitive skills appropriately.

This literature review and meta-analysis confirms that the executive functions display greater predictive power at early ages and have a robust, specific capacity for predicting future academic performance. Thus, it is important to detect academic achievement problems as early as possible to initiate intervention programs. The intent would be to minimize any potential problems that are inherent in learning, particularly those that hinder normal development in language and mathematics. This is confirmed by some of the longitudinal studies reviewed here such as those by Aarnoudse-Moens et al. (2013), Alloway and Alloway (2010), Hall et al. (2015), Oberer et al. (2018), and Welsh et al. (2010). However, in some cases, the relationship patterns between these variables are sustained throughout the longitudinal study for all of the various age groups (Oakhill et al., 2011). Other studies determined that normal, early childhood development helps students who begin their schooling late catch up to the rest of the students (Ribner et al., 2017). Of note are the studies that begin their research in the pre-school stage and conduct follow-ups over 3 to 6 years, with the objective of predicting academic performance in primary school. Best et al.'s (2011) article on a study of children from 5 to 17 years old (the broadest age range) determined that the most intense development of executive functions occurred among the youngest children. It then slowed somewhat in the last years of infancy and declined during adolescence. That study also demonstrated a direct relationship between this variable and academic performance as well as an indirect relationship through the verbal factor and logical reasoning. This connection indicates the link that executive components such as working memory, inhibition and attention have with mathematical competence and language development. On the contrary, Bryce et al. (2015) used a specific structured model based on the moderation of variables and concluded that after the age of seven, executive functions and cognitive abilities decline dramatically in the subsequent 5 years. Despite this discrepancy, our meta-analysis suggests that executive functions are essential for the development of academic skills in primary school.

Characteristics such as age, gender, socioeconomic status, and physical fitness can act as moderators in the relationship between executive functions and academic performance, as shown in previous research (see Thomson, 2018; Kvalø et al., 2019). In the current meta-analysis, two meta-regressions were performed—one for age and one for gender—and no significance was found for the first (age), and a 49% variance was found for the second (gender). A possible explanation for these findings is that during this age range, females mature more rapidly than males. The 7 to 12 age range corresponds to a period of cognitive transition that Piaget (1991) calls the concrete operational stage. Children can make logical inferences and reversible mental operations, and they can formulate hypotheses. In this stage, the reinforcement of mnesic processes and metacognition occurs (memory, knowledge, learning strategies, the monitoring of one's own thoughts, semantic elaboration). It is precisely in this educational period when gender differences between boys and girls become the basis for the diverging cognitive development of the genders. Different aptitudes, behaviors and abilities emerge (Calvo, 2009). Kovacs and Devlin (1998) make a number of observations on this topic: there is a different rhythm of physical, cognitive and psychic maturation for men and women. Females mature at an earlier age, which produces disparities in learning and academic performance. Consequently, females display better writing skills during the first years of school (due to the development of fine motor skills) as well as better verbal skills and abilities. Males have the advantage of better visuo-spatial capabilities due to the effects of testosterone.

There have been numerous studies that examine the differences in academic performance between the genders. Hyde et al.'s (1990) meta-analysis compiled hundreds of studies on the influence of gender on academic performance. Half of the studies showed very minor differences, and a third of the studies found no differences at all. The widely held acceptance of the disparate cognitive abilities of men and women was broken down, and it was suggested that social and cultural factors influenced performance in the various academic fields. There is no evidence that boys are better at mathematics and girls are better at language. The study by Hyde and Mertz (2009) aligns with the one by Hyde et al. (1990) as it concludes that girls achieve the same results as boys in standardized math tests. In addition, there is no difference in language ability between men and women. Other studies, such as those by Alcaraz and Guma (2001) or Mathiesen et al. (2013), contend that in addition to the divergence that comes from sexual dimorphism, there are differences in brain anatomy such as the larger corpus callosum in females, which facilitates the processing of language, and a larger nucleus of the hypothalamus in males that influences emotions. The latter author determined that the literature on the cognitive development of the two genders presents different results depending on age, the time period, and location. Along this same line, Bethencourt and Torres (1987), Herrera et al. (2000), and Steinmayr and Spinath (2009) determined that girls start school with significantly higher levels of lexical and motor skill development than boys. This could be due to their earlier maturation, which can present differences of almost 2 years during puberty. These authors also assert that overall performance by females is on average slightly higher than male performance during their first years of school. In addition, they note that females have better inhibitory control; however, no gender-based differences in the processes of cognitive flexibility were noted. In the early years of primary education, girls demonstrate better results for working memory, short-term memory and attention. In the later years of primary education, due to age and years of schooling, they achieve a good level of execution, categorization and conceptualization (Reyna and Brussino, 2015). These results, which are better for girls than for boys, will be related to the language capabilities attributed to left-hemisphere brain development, which is delayed in males due to the presence of testosterone (Acosta, 2001). Therefore, the existing literature is completely consistent with the results of our meta-analysis, in which gender emerges as a moderating variable between academic performance and the executive functions during the primary school years.

It is important to note that in the conducted meta-analysis, there are two articles with outlier data. However, this is not due to a bias error but to a real variability in the variance and effect size. This result is due to the particular research design and the treatment of the academic performance variable. In some cases, as in the article by de Bruijn et al. (2018), this variable was studied from the opposite perspective of poor performance. These studies present out-of-range effect sizes: *r* = 0.14 (de Bruijn et al., 2018) and *r* = 0.137 (Mulder et al., 2017). The study by de Bruijn et al. (2018) also present confidence intervals (95% CI) that contain zero, which annuls any statistical significance in the relationship. The same does not occur in the study by Mulder et al. (2017); however, it contains parameters below those established in this meta-analysis (an effect size of *r* = 0.365, with a 95% CI between 0.309 and 0.419 in the relationship between executive functions and academic performance). These studies were conducted in the Netherlands, and in all two there is a variability of the variance that points directly to language and to the distinctiveness of the design or sample. In one of them, works with two groups in which 25% of the sample corresponds to children with poor academic performance (de Bruijn et al., 2018). The last study has the distinct feature of being a longitudinal design that starts with 3 year olds (Mulder et al., 2017). Despite this distinction, no differences were observed in the behavior of the executive function components in relation to language, regardless of the culture, the native language, or the educational system where the research was conducted.

## Conclusions and Limitations

Although there are many publications on academic performance and the variables that influence it, the originality of this meta-analysis lies in its focus on the last decade and on primary education. In addition, this study considered and included a variety of samples and research studies conducted in different countries to provide a comprehensive overview of the topic. The publication review made it possible to verify the diversity of the variables related to academic performance, highlighting the executive functions. The primary education stage was the focus of only a small number of studies, compared to the pre-school or secondary education stages; the university stage was studied the most. The number of competency-based measures of academic performance in the studies increased with a corresponding decrease in the use of numerical grades (per quarter and subject), which is considered to be closer to a true measure of learning. There was an increased number of studies with structured models using first-level variables such as the executive functions and other variables deemed to be minor because they are influenced by the former (not because of their direct relationship but because of their moderating power); however, they are also essential for the development of certain competencies and capabilities.

An important finding is that it was possible to confirm that, in the last decade, executive functions have replaced the intelligence quotient as the most studied variable with respect to academic performance and that both currently have the same predictive capacity. The results of this review and meta-analysis support the recognition of the multifactorial composition of executive functions, and they reveal that working memory is the most researched component as well as a better predictor than inhibition. In addition, it is evident that the behavior of the executive function components depends on the subject studied, especially regarding the relationship between mathematics and the visuo-spatial aspect of working memory. Similarly, most of the executive components are better related to performance in mathematics than in language. Given the dilemma of classifying executive functions as a domain-general cognitive variable, the studies reviewed confirm that executive functions can be decomposed into different components (working memory, inhibition, cognitive flexibility and planning) that are distinctly linked to certain types of learning. Furthermore, the moderating role of executive functions was demonstrated with respect to other variables such as physical fitness, motor skills, or memory processes. Similarly, it is evident that the executive functions are an important predictor of academic performance and future learning problems at an early age. However, this variable diminishes in its predictive capacity during secondary education and more so during university-level education where its development cycle comes to an end. Deficiencies detected in the executive components affect levels of school performance, which in turn has a heavy influence on the subsequent development of people at all levels—training, employment, social life. There is another important finding that must be highlighted: the moderating effect of gender in the relationship between executive functions and academic performance. The explanation is found in the significant maturational development that occurs during the years of primary education. Due to physiological and neurological factors, girls mature more quickly than boys during this stage. The studies reinforce the descriptive and moderating nature of this variable with respect to the development of the various skills needed for acceptable learning in primary school, in addition to its link to the student's maturity.

Since our meta-analysis included studies from different continents, from different socioeconomic levels, and from different rural or urban areas, it indirectly addressed the impact that different educational systems can have on intellectual development. However, no significant differences were found that could have produced variability in the executive component resulting from the sociocultural and educational contexts of the samples. In this regard, the diverse measures of academic performance, expressed in the (mostly) nationwide standardized achievement test results and in the traditional numerical grades given by teachers for the various subjects, have not demonstrated any significant differences. All this indicates that culture, native languages, socioeconomic levels, and the various objective methods of assessing this variable do not affect its development nor the resulting statistical data.

With regard to the limitations of this study, its sampling and research design can be noted. The descriptive and correlational nature of the study meant that the only statistics included were those that directly related to the variables studied and that those that compared groups or established indirect relationships were excluded. If these are linked to the results of our systematic review and the conclusions reached, then future research should consider focusing on the specific nature of the executive functions, using as a reference the statistics from the various structured models as well as their connection to the development of specific capabilities and competencies. Moreover, once the importance of maturity on the development of executive functions has been proven, we suggest a study on the relationship between the two, focusing on gender and not exclusively on age. All this can contribute to the development of specific intervention plans for the executive function components and deficient capabilities that can guide efforts to improve the learning process for students. They can also contribute to furthering the understanding of the links between this variable and academic performance at an early age.

## Author Contributions

AQ conducted the search, selection, and coding of the research articles. He also conducted the statistical analyses and drafted the initial draft of the manuscript. AC and NM reviewed the coding of the selected articles and reviewed and corrected the initial draft of the manuscript. AC did the coordination work and all three approved the final manuscript submitted.

### Conflict of Interest Statement

The authors declare that the research was conducted in the absence of any commercial or financial relationships that could be construed as a potential conflict of interest.
